# Clinical efficacy of phacoemulsification combined intraocular lens implantation for treatment of high myopia with cataract: A protocol of systematic review

**DOI:** 10.1097/MD.0000000000023215

**Published:** 2020-12-04

**Authors:** Lu Gao, Mei Li

**Affiliations:** Department of Ophthalmology, Yan’an City Hospital of TCM, Yan’an, China.

**Keywords:** high myopia, cataract, phacoemulsification, intraocular lens implantation

## Abstract

**Background:**

The purpose of this study is to assess the clinical efficacy of phacoemulsification combined intraocular lens implantation (PILI) for the treatment of high myopia with cataract (HMC).

**Methods:**

In this study, the electronic databases (PUBMED, EMBASE, Cochrane Library, Scopus, AMED, CINAHL, PsychINFO, CBM, and China National Knowledge Infrastructure) will be searched from inception to present. All randomized controlled trials on assessing the PILI for patients with HMC will be included. Two authors will carry out study selection, information extraction, and study quality assessment, respectively. We will invite another author to solve any disagreement through discussion. RevMan 5.3 software will be performed for data synthesis and analysis.

**Results:**

This study will present a detailed synthesis and/or descriptive analysis of the most recent evidence to evaluate the efficacy of PILI for HMC.

**Conclusion:**

The results of this study may provide possible guidance to determine whether or not PILI is effective on HMC.

## 1 Introduction

High myopia is a common disorder globally, that affects almost the whole eye.^[1–4]^ It is associated with high refractive errors, open-angled glaucoma, retinopathy, and cataract.^[2,5–10]^ Its global prevalence was about 163 million patients in 2000, which accounted for 11.6% of all myopia patients, and 2.7% of population around the world.^[11,12]^ It has been estimated that its prevalence will be increased to 938 million by 2050, which will yield 9.8% of global population, and 19.7% of all myopia subjects.^[11,13]^ Patients with HM tend to develop cataracts more easily than normal eyes.^[14–17]^ Thus, it is very tricky to treat high myopia with cataract (HMC).^[17–19]^

A variety of studies have reported that phacoemulsification combined with intraocular lens implantation (PILI) can be used to treat HMC.^[20–31]^ Although evidence of PILI for HMC is promising, there is no consensus on the PILI for HMC, and much of the literature had been no comprehensive assessment of its efficacy and safety. This study aims to critically appraise the efficacy of PILI for HMC with an evaluation of the quality of the available trials.

## 2 Methods

### 2.1 Study registration

This study was registered on OSF (osf.io/6mkjx). Its report follows the guideline of Preferred Reporting Items for Systematic Reviews and Meta-Analysis Protocol statement.^[32]^

### 2.2 Criteria for eligible studies

#### 2.2.1 Study types

This proposed study will consider randomized controlled trials (RCTs) on exploring the efficacy of PILI on HMC. We will exclude any other study, except RCTs.

#### 2.2.2 Participant types

All eligible patients who were diagnosed as HMC will be included in this study, regardless of the race, sex, age, and severity of HMC.

#### 2.2.3 Intervention types

In the intervention group, all patients underwent PILI as only intervention.

In the control group, all patients could receive any therapy, except for any forms of PILI.

#### 2.2.4 Outcome types

Outcomes include mean axial length, mean intraocular lens, visual acuity, intraoperative pain, ocular discomfort, quality of life, and adverse events.

### 2.3 Strategy of literature searches

Major searches were performed in electronic databases (PUBMED, EMBASE, Cochrane Library, Scopus, AMED, CINAHL, PsychINFO, CBM, and China National Knowledge Infrastructure) from inception to present. We will not place any limitation to language and publication time. All search strategies will be created by a professional librarian in collaboration with study team. Exemplary search strategy of PUBMED is shown in Table 1. Similar search strategy will be adapted to other electronic databases.

**Table 1:** Search strategy of PUBMED.

Number	Search terms
1	Myopia
2	Short-sightedness
3	Nearsightedness
4	Visual function
5	Eyesight
6	Impaired vision
7	Cataract
8	Vision loss
9	Clouded vision
10	Blurred vision
11	Dim vision
12	Double vision
13	Or 1–12
14	Phacoemulsification
15	Cataract surgery
16	Ultrasound probe
17	Intraocular lens
18	Implantation
19	Operation
20	Or 14–19
21	Random
22	Randomly
23	Randomization
24	Blind
25	Placebo
26	Sham
27	Control
28	Clinical
29	Trial
30	Study
31	Or 21–30
32	13 and 20 and 31

In addition, we will also search other sources, such as dissertation/thesis, conference abstracts, and reference lists of included studies.

### 2.4 Data extraction

#### 2.4.1 Study selection

All searched citations will be imported into EndNote X9, and titles/abstracts of those citations will be scanned to eliminate unrelated and repetitive studies. Then, the full text of potential articles will be further judged based on the inclusion criteria. The whole process of study selection will be rendered in a flowchart. Two authors will independently perform study selection. Any disagreement will be settled by another author through discussion.

#### 2.4.2 Data extraction

Two authors will independently extract information from each included trial, including general information (such as study title, first author, year of publication), patient information (such as demographics, diagnostic criteria, inclusion and exclusion criteria), study setting, study methods, interventions, controls, outcome indicators, results, conclusion, adverse events, and funding information. If there are differences between 2 authors, we will consult another experienced author and a final decision will be reached.

#### 2.4.3 Dealing with missing data

For missing or unclear data, we will contact corresponding authors via email or phone to obtain that information. If we can not achieve those data, we will analyze available data using intention-to-treat analysis.

### 2.5 Study quality assessment

Two authors will independently appraise study quality using Cochrane Collaboration Tool. It will assess each study through 7 aspects, and each item is appraised at three levels: low risk of bias, unclear risk of bias, and high risk of bias. Any difference will be solved by discussion with the help of another author.

### 2.6 Statistical analysis

This study will utilize RevMan 5.3 software for statistical analysis. Treatment effect of continuous values will be calculated as mean difference with 95% confidence intervals. Treatment effect of dichotomous values will be expressed as risk ratio with 95% confidence intervals. Statistical heterogeneity across eligible studies will be detected by *I*^*2*^ test. We will employ a fixed-effects model to pool the data if *I*^2^ ≤ 50%. We will conduct a meta-analysis if sufficient data are collected, and adequate similarity in study information, patient characteristics, study methods, and details of intervention and control on the same outcome are detected. On the other hand, we will place a random-effects model to synthesize the data if *I*^2^ > 50%. we will carry out a subgroup analysis to explore its causes of substantial heterogeneity. If data can not be pooled, we will report outcome results by narrative description.

### 2.7 Additional analysis

If remarkable heterogeneity is detected across eligible studies, we will carry out a subgroup analysis according to different study information, patient characteristics, study quality, details of intervention and control, and outcome measurements.

Sensitivity analysis will be conducted to examine the robustness of pooled outcomes by getting rid of low quality studies.

We will investigate reporting bias by funnel plot^[33]^ and Egger regression test^[34]^ if over 10 RCTs are included in this study.

### 2.8 Ethics and dissemination

Ethical approval is not needed in this study, because it will only analyze published data. We will plan to publish this study on a peer-reviewed journal.

## 3 Discussion

HMC is one of the most common diseases in the ophthalmology clinic. If it can not be treated fairly well, it may bring a variety of adverse events, such as blind. Currently, the available clinical treatments of HMC include medication, surgery, and alternative therapies.^[16–19]^ However, their efficacy is still unsatisfied. At present, there are many reports that PILI has a significant efficacy on HMC,^[20–31]^ but its efficacy and safety is still uncertain. So far, no systematic review on this topic has been reported. Therefore, it is necessary to undertake a high-quality study to systematically assess the published RCTs of HMC for PILI and to provide helpful evidence for its clinical practice.

This study may have several limitations. First, this study may fail to include an adequate number of eligible RCTs. Second, the heterogeneity of PILI may be variations due to the different forms of PILI. Third, smaller sample size of included trials will result in higher risk of bias.

## Author contributions

**Conceptualization:** Lu Gao, Mei Li.

**Data curation:** Lu Gao, Mei Li.

**Formal analysis:** Lu Gao, Mei Li.

**Investigation:** Mei Li.

**Methodology:** Lu Gao, Mei Li.

**Project administration:** Mei Li.

**Resources:** Lu Gao.

**Software:** Lu Gao.

**Supervision:** Mei Li.

**Validation:** Lu Gao, Mei Li.

**Visualization:** Lu Gao, Mei Li.

**Writing – original draft:** Lu Gao, Mei Li.

**Writing – review & editing:** Lu Gao, Mei Li.
